# Mechanical and Geometric Performance of PLA-Based Polymer Composites Processed by the Fused Filament Fabrication Additive Manufacturing Technique

**DOI:** 10.3390/ma13081924

**Published:** 2020-04-19

**Authors:** José María Reverte, Miguel Ángel Caminero, Jesús Miguel Chacón, Eustaquio García-Plaza, Pedro José Núñez, Jean Paul Becar

**Affiliations:** 1 Escuela Técnica Superior de Ingeniería Industrial, IMACI, Universidad de Castilla-La Mancha, Campus Universitario s/n, 13071 Ciudad Real, Spain; josemaria.reverte@uclm.es (J.M.R.); jesusmiguel.chacon@uclm.es (J.M.C.); 2 Escuela Técnica Superior de Ingeniería Industrial, INEI, Universidad de Castilla-La Mancha, Campus Universitario s/n, 13071 Ciudad Real, Spain; eustaquio.garcia@uclm.es (E.G.-P.); pedro.nunez@uclm.es (P.J.N.); 3 Laboratoire de Mathématiques et leurs Applications de Valenciennes, University Polytechnique Hauts-de-France, FR CNRS 2956, F-59313 Valenciennes, France; jean-paul.becar@uphf.fr

**Keywords:** 3D printing, Fused Filament Fabrication, mechanical characterization, fibre-reinforced PLA composites, dimensional accuracy, surface texture

## Abstract

In this work, the effect of short carbon fibre (CF) on the mechanical and geometric properties of 3D printed polylactic acid (PLA) composite parts processed using the Fused Filament Fabrication (FFF) technique have been analysed. Tensile, flexural and interlaminar shear strength (ILSS) tests were performed to obtain the mechanical performance of the different samples. The surface quality and geometric accuracy of the printed specimens were also evaluated. Finally, Scanning Electron Microscope (SEM) images of the printed samples are analysed. The results revealed that the addition of carbon fibres effectively improved all assessed mechanical properties of PLA-CF composites as compared to the neat PLA. In particular, Flat PLA-CF samples showed an average increase in tensile performance of 47.1% for the tensile strength and 179.9% for the tensile stiffness in comparison to the neat PLA. From the flexural behaviour point of view, Flat PLA-CF samples revealed an increase in average flexural strength and stiffness of 89.75% and 230.95%, respectively in comparison to the neat PLA. Furthermore, PLA-CF samples depicted the best ILSS performance. In general, the use of short carbon fibre as reinforcement did not affect the dimensional accuracy of the PLA-CF samples, and even improved the surface roughness in certain cases, particularly in Flat and On-edge orientations.

## 1. Introduction

Additive manufacturing (AM) is a very promising technology permitting the development of 3D printed models or functional parts with complex geometries. Fused Filament Fabrication (FFF) is a widely adopted AM technique due to its simplicity, low cost and material wastage [1,2,3,4,5,6]. The FFF technique generates a 3D object through the deposition of a thermoplastic extruded filament such as polyamide (Nylon), acrylonitrile butadiene styrene (ABS) or polylactic acid (PLA). PLA is in great global demand due to its application in packaging, automotive, pharmaceutical and textile engineering [7,8]. In addition, PLA is an eco-friendly and biocompatible material with good physical properties, making it ideal for biomedical applications [9].

However, the FFF technique has been traditionally restricted to rapid prototyping applications due to the poor mechanical and geometrical properties of the printed parts, since pure polymers products developed by FFF lack the sufficient strength for use in functional parts [10]. Additionally, injection-moulded components typically have higher mechanical properties than FFF printed parts of the same thermoplastic [11].

In recent years, FFF has started to move beyond the prototyping stage to the fabrication of finished functional components, topology optimized parts [12] or moulds for composite materials and structures [13] that must comply with structural and quality requirements. This has been achieved by the development of new reinforced composites with improved mechanical properties, and improvements in the accuracy of 3D printing machines. 

Regarding AM material, the addition of particles or fibres as reinforcement into polymers enables the manufacturing of composites that exhibit high mechanical performance with improved functionality [14]. Fibre reinforced composites can be obtained by embedding short or continuous fibres into the thermoplastic matrix. Short fibres such as chopped carbon, glass or aramid fibres, have been analysed in several studies with a moderate improvement in mechanical properties [15,16,17,18,19,20]. In most studies, short fibres were embedded in PLA, ABS or Nylon thermoplastic filaments prior to loading in the 3D printer. In addition, the use of continuous fibres such as carbon, glass or Kevlar fibres has been examined by several groups [10,21,22,23,24,25]. Recently, a limited number of studies have reported the development of 3D printed composites using graphene-based particles as reinforcement [6,26,27,28]. 3D printed continuous fibre reinforced composites show higher mechanical performance than 3D printed short fibre reinforced composites. For example, the tensile properties of 3D printed continuous carbon fibre reinforced composite samples were roughly an order of magnitude higher than 3D printed short fibre or particle reinforce composites [29]. Thus, 3D printed continuous fibre reinforced composites are more suitable for manufacturing functional parts for advanced applications [22,29,30]. However, the processing requires a specially designed printer [22]. Two main options for the fabrication of continuous fibre reinforced composites have been analysed in previous studies: a “co-extrusion” process of continuous fibre and polymer matrix in the injector and a “dual extrusion method” of continuous fibre and polymer matrix directly into the component [21]. Furthermore, the use of short fibre reinforcement enables higher quality parts in terms of geometric performance, with lower porosity than the continuous ones. The use of short carbon fibre reinforcements has allowed for the extensive development of AM, since it improves the mechanical and dimensional properties of printed parts as compared to pure thermoplastic materials, without significantly increasing manufacturing costs or modifying the 3D printing methodology [13]. In addition, there is a dearth of studies on the interlaminar bonding behaviour of PLA-based composites. The microstructure, and consequently the mechanical performance of the PLA-based composite parts, are significantly affected by the bounding quality between deposited strands and layers [16,31]. 

Moreover, the geometric quality of FFF 3D printed parts has received less attention in the literature, than the study of its mechanical properties [32]. The geometric quality supposes a great limitation of FFF processes and that is why traditional manufacturing processes are preferred over this incipient technology when high quality products are required. Thus, an improvement of dimensional accuracy [33,34,35], and surface texture [36,37] is a significant issue in FFF processes. The geometric characterization has mainly focused on conventional polymers without any reinforcement [38,39], whilst studies on composite parts are somewhat scarce [6,26,40]. Composite materials not only improve the mechanical properties, but also the geometric characteristics. For this reason, further studies are required to analyse the geometric performance of additive manufactured PLA-based composite parts.

In this study, commercially available PLA and carbon fibre reinforced PLA composite (PLA-CF) parts were manufactured using a FFF low-cost desktop printer. The objective of this study was to assess the effects of short carbon fibre reinforcement on the mechanical and geometric behaviour of 3D printed composite samples. In particular, the effect of build orientation was evaluated. Tensile, flexural and interlaminar shear performances was examined to obtain the mechanical behaviour of the different samples. Finally, SEM images of the 3D printed samples were appraised to examine the effects of reinforcement on fracture and geometric performance.

## 2. Materials and Methods

### 2.1. Material, 3D Printing System and Specimen Fabrication

**Figure 1:**
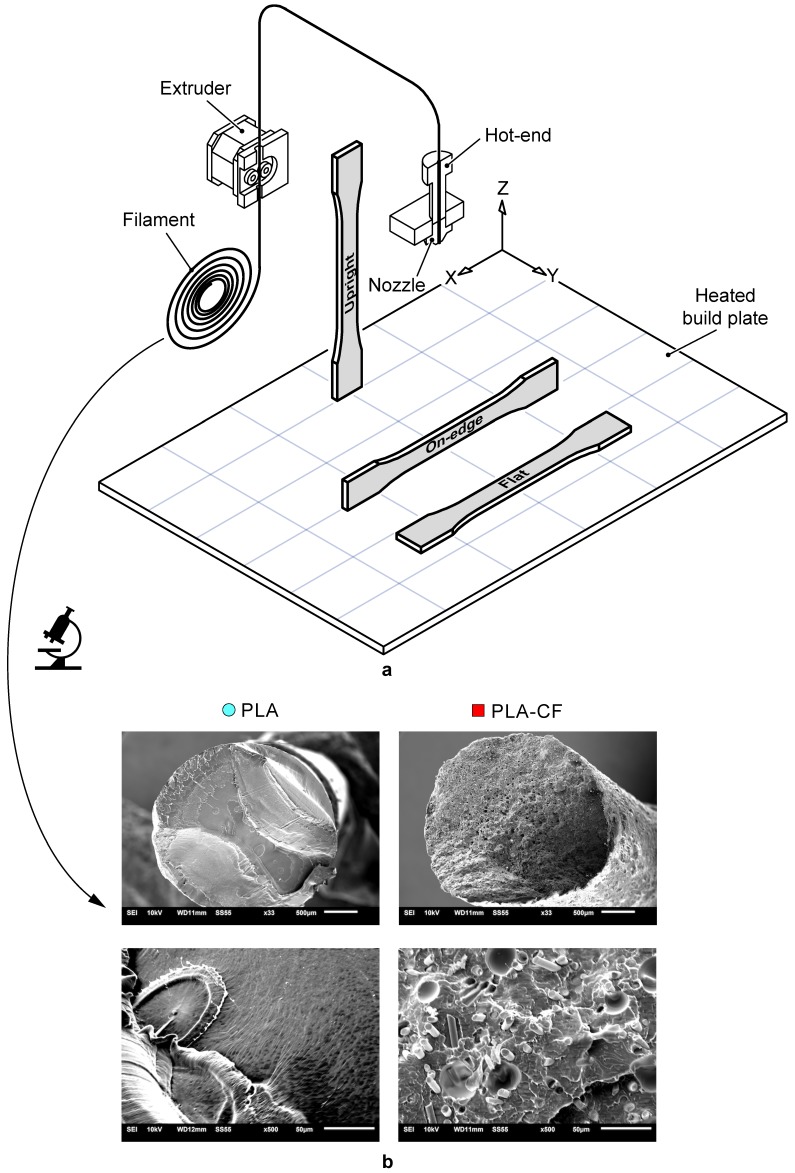
(**a**) Sketch of the Ultimaker 2+ 3D printer and build orientation. (**b**) Cross sectional SEM images of printing wires: PLA and PLA-carbon fibre (CF).

In this study, two different commercially available PLA-based filaments have been analysed: SMARFIL^®^ PLA natural (PLA) [41], and CarbonX™ filament, a PLA composite filament reinforced with short carbon fibres (PLA-CF) manufactured by 3DXTech (Grand Rapids, MI, USA) [42]. CarbonX™ Carbon Fibre Reinforced polylactic acid (PLA) is a high-performance carbon fibre reinforced 3D printing filament. This PLA composite was formulated using high-modulus carbon fibre and premium Natureworks PLA biopolymer. CarbonX™ Carbon fibre reinforced-PLA is ideal for applications demanding greater stiffness and dimensional stability than traditional unfilled materials. The basic mechanical properties of the PLA and PLA-CF filaments provided by the manufacturer [41,42] are presented in Table 1. In addition, Figure 1 depicts SEM images of the cross-sectional surfaces of the PLA and the PLA-CF filaments. SEM images were taken using gold sputtering by a JEOL LTD JSM-6610LV scanning electron microscope (Peabody, MA, USA). The detailed presence of uniformly distributed carbon fibre reinforcements embedded in the polymeric PLA matrix can be seen, and some pores due to fibre pull-out during testing.

PLA and PLA-CF samples were manufactured using a Ultimaker 2+ desktop 3D printer made by Ultimaker (Ultrecht, Netherlands) [43]. This 3D printer has interchangeable nozzles and a heated build plate that allows the use of a large variety of materials. The 3D printing system consisted of an extruder, temperature-control system, a heated build plate, X–Y–Z motion mechanism and a heated print head with different nozzle diameters. The details of the different elements of the 3D printer system are shown in Figure 1. Table 2 shows the technical specifications of the Ultimaker 2+ 3D printer. The specimens were modelled using the commercially available CAD system SolidWorks (2018-2019), exported as a stereolithography file (STL) with fine resolution and a deviation of 0.0171 mm and imported by the 3D printing software. The G-code files were generated using the Ultimaker Cura 3D software (4.3.0) [44].

**Figure 2:**
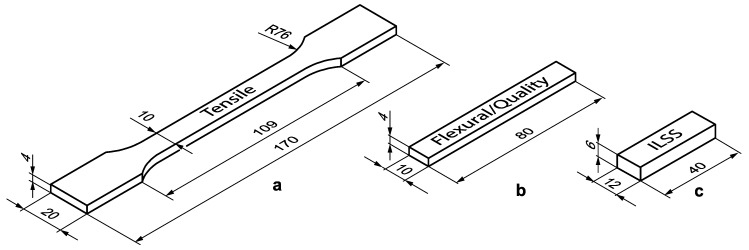
Shape and dimension of specimens for (**a**) tensile, (**b**) flexural and dimensional and (**c**) interlaminar shear strength (ILSS) tests. Dimension are in mm.

Figure 2 shows the dimensions of the samples based on the recommendations of the ASTM standards D638, D790 and D2344 for testing the tensile, flexural and interlaminar shear performance of samples, respectively. For dimensional tests, flexural specimens have been employed. 

### 2.2. 3D Printing Settings

The selection of FFF printing settings determining the mechanical and geometric performance of the manufactured parts [3,45]. Bearing this in mind, the main aim of this study was to analyse the effect of the type of reinforcement and build orientation on PLA-based composites. Hence, different FFF printing settings were fixed for all the samples (Table 3). Three build orientations were analysed (Figure 1): Flat (F), On-edge (O) and Upright (U). In Flat and On-edge orientations, the tensile pull direction was parallel to the X-axis. However, in the Upright orientation, the tensile pull direction was parallel to the Z-axis. Five specimens for each orientation and material were prepared.

### 2.3. Experimental Set-Up

Mechanical characteristics of PLA-based samples were measured by uniaxial, 3-point bending and ILSS tests. These tests were conducted according to ASTM standard recommendations, using a universal testing machine (Madrid, Spain) equipped with a load cell of 5 kN. A high-performance axial extensometer MTS 634.14 (Minnesota, USA) was employed to measure the tensile strain. The tensile and flexural stress–strain curves were obtained with a constant loading rate of 2 mm/min. ILSS tests were conducted with a constant loading rate of 1 mm/min. Dimensional accuracy was measured using an optical measure machine vision system Tesa-Visio 200 (Renens, Switzerland), with a resolution of 0.001 mm. A Talysurf CLI 1000 profilometer (Leicester, England) with a Z axis resolution of 40 nm was employed to measure the texture of samples. More information of the experimental details can be found in the authors’ previous works [6,32].

## 3. Results and Discussion

The following sections show a detailed discussion of the main effects of the type of reinforcement and build orientation on the mechanical and geometric performance PLA-based composite parts.

### 3.1. Effect of Carbon Fibre Reinforcement and Build Orientation on the Mechanical Behaviour of PLA Composite Parts

#### 3.1.1. Tensile and Flexural Properties of PLA Composite Samples

**Figure 3:**
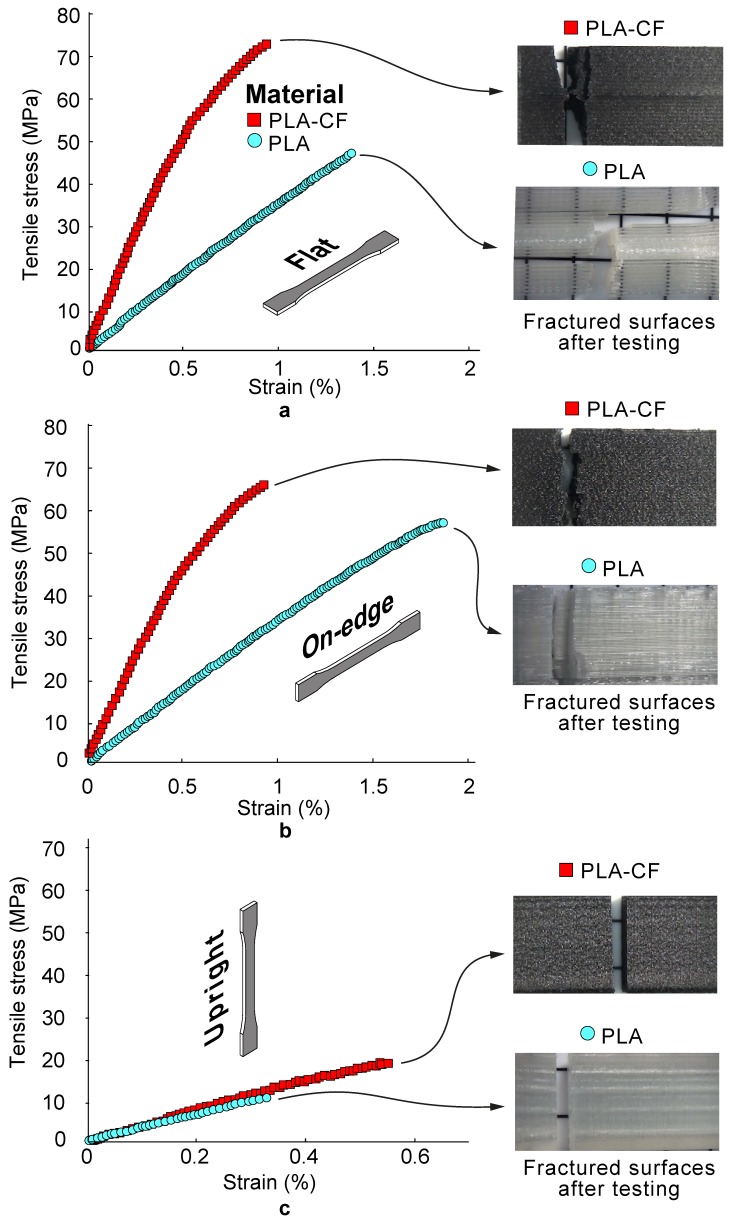
Average tensile stress–strain curves of PLA and PLA-CF 3D printed samples for (**a**) Flat, (**b**) On-edge and (**c**) Upright orientation. Optical micrographs showing details of the fractured surfaces after testing are included for comparative purposes.

**Figure 4:**
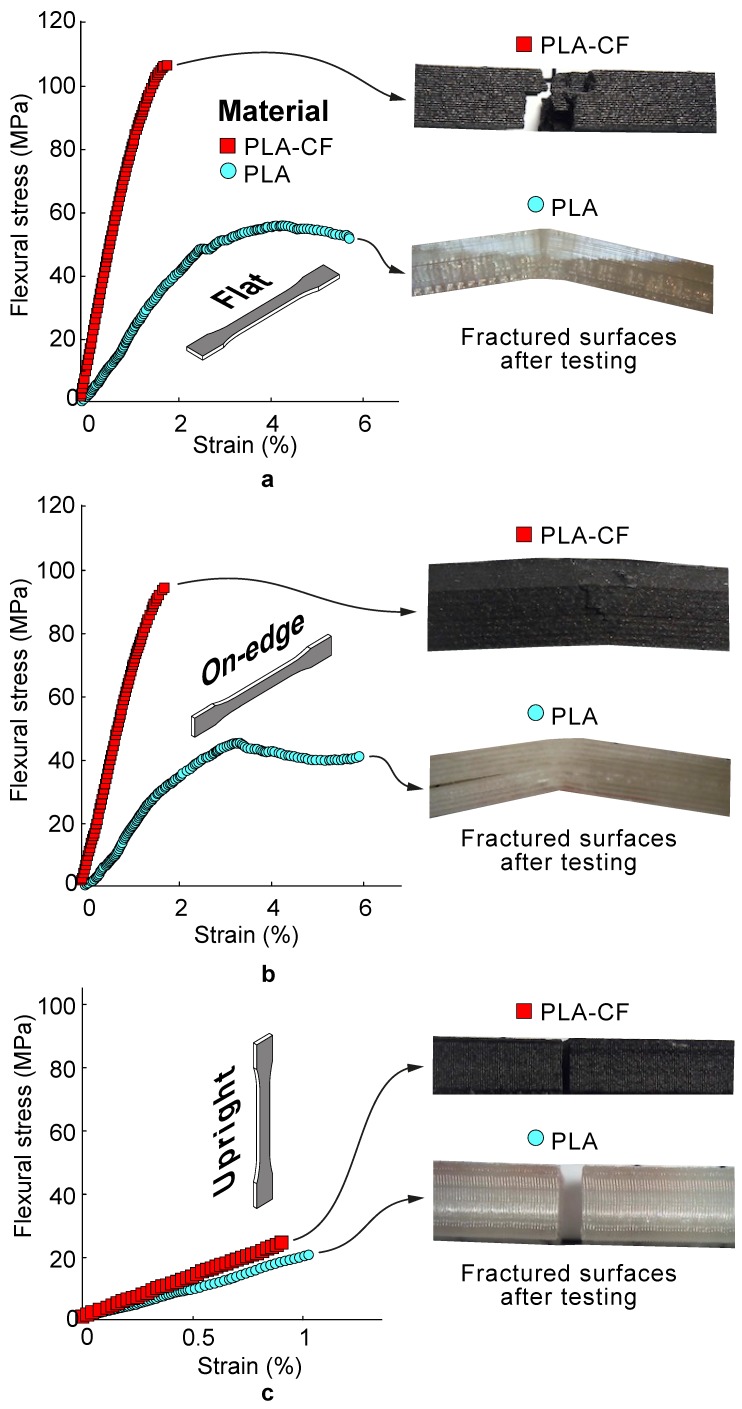
Average flexural stress–strain curves of the PLA and PLA-CF 3D printed samples for (**a**) Flat, (**b**) On-edge and (**c**) Upright orientation. Optical micrographs showing details of the fractured surfaces after testing are included for comparative purposes.

**Figure 5:**
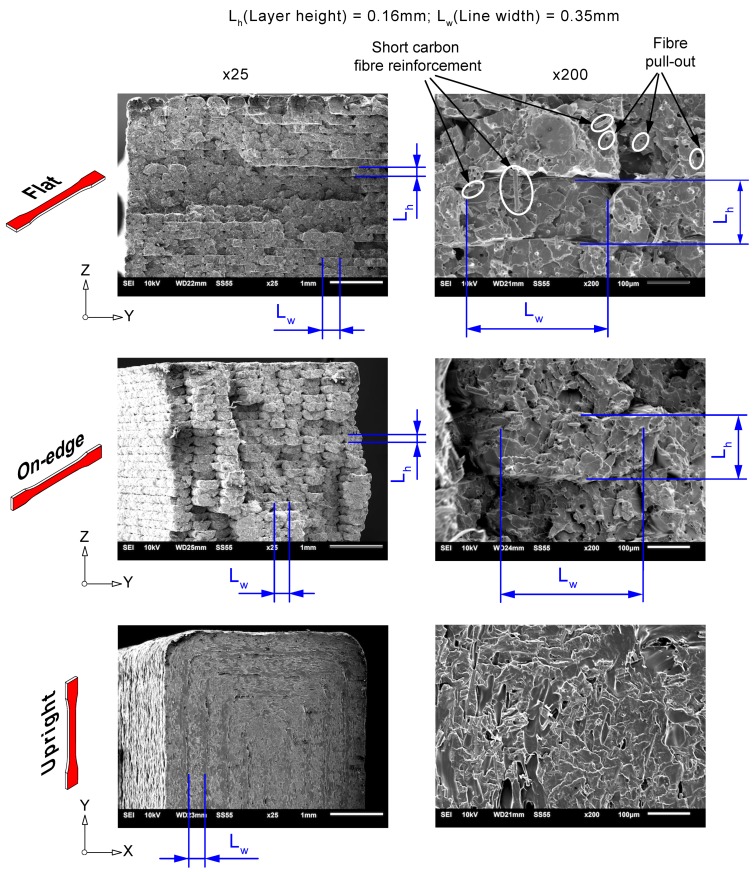
SEM images revealing details of the cross-sectional tensile fracture surfaces of 3D printed PLA-CF composite samples as a function of the build orientation. Details of the morphology of the deposited strands are included for comparative purposes. *L_h_* and *L_w_* are the layer height and the deposited strand width, respectively.

Table 4 shows the experimental results of the tensile and flexural performances in terms of average stress and stiffness. Figure 3 and Figure 4 report some representative tensile and flexural stress–strain curves of PLA composite samples for different build orientations. Additionally, Figure 5 includes SEM images revealing details of the cross-sectional tensile fractured surfaces. From Table 4 and Figure 3, Figure 4 and Figure 5 it can be seen that the 3D printed PLA-based composite samples showed a remarkable anisotropy. It has been found that the mechanical properties depended significantly on the build orientation. Firstly, the effect of build orientation was analysed. On-edge and Flat orientations exhibited the highest values for both tensile and flexural strengths and stiffness, while Up-right orientation exhibited the lowest ones. The differences between Flat and On-edge in the mechanical performance were lower than 8%. However, Flat PLA and PLA-CF parts depicted an increase in tensile strength of 484% and 386% compared to Upright PLA and PLA-CF composites specimens, respectively. Overall, the results showed a more ductile behaviour for the Flan and On-edge orientations, with higher plastic deformation. However, Upright orientation showed a brittle behaviour. In general terms, the best mechanical performances have been obtained with Flat orientation.

Secondly, the effects of the addition of carbon fibre reinforcement were analysed. In comparison to the pure Flat PLA polymer, the tensile strength of the Flat PLA-CF composite increased by 47.1% from 47.8 MPa to 70.3 MPa. This result can be attributed to the remarkable strength and modulus of carbon fibre reinforcement given that the mechanical performance of composites normally can be enhanced via the incorporation of superior reinforcement with higher stiffness than the matrix.

Similar with the influence of carbon fibre reinforcement on strength, PLA-CF samples exhibited greater stiffness E_t_ than PLA samples for each of the three orientations. Flat PLA-CF samples obtained an average increase in tensile modulus of 179.9 % compared to Flat PLA samples, from 3.35 GPa to 9.21 GPa. Carbon fibre reinforcements prevented the shear strain as they provide greater stiffness than the PLA matrix. These results are in agreement with previous studies [6,46]. This increased stiffness E_t_ suggested that the reinforced samples showed greater resistance to plastic deformation due to the effective load transfer to the carbon fibres. However, PLA-CF samples showed lower strains at failure, denoting a more brittle behaviour than that in the PLA samples. 

Furthermore, the assessment of flexural properties (i.e., strength and stiffness) for composites is crucial to avoid potential deflection and bending fractures when subjected to high loading and sliding velocity during some engineering applications. In view of this consideration, flexural tests were conducted to compare the PLA samples with PLA-CF samples via the three-point bending method. As shown in Figure 4, both the flexural strength and modulus for neat PLA and PLA-CF composites exhibited the same trends as those observed for tensile characterization. Flat PLA-CF samples showed an average increase in tensile strength and stiffness of 89.75% and 230.95% in comparison to Flat PLA specimens, respectively. These improvements in flexural performance (especially for the modulus) were due to the addition of high stiffness carbon fibre reinforcements. 

In addition, SEM examination of the cross-sectional tensile fractured surfaces of 3D printed PLA-CF specimens is depicted in Figure 5. This study was performed to gather data on the effects of the reinforcement and build orientation on the morphology of deposited strands. Overall, the results depicted a brittle behaviour for the upright orientation, with the fracture surface contained on a plane. However, On-edge and Flat orientations showed greater plastic deformation. Different fracture planes were observed with extensive fibre pull-out. Moreover, details of the cross-sectional shape of the fused deposited strands, their periodicity and the triangular shaped inter-filament voids between deposited strands were observed. According to the layer-by-layer deposition technique of the FFF process, the melted filament is extruded through the nozzle and squeezed onto the printing bed. Since the deposited filament is still soft from heat of extrusion, the lower part was then flattened, and the top part exhibited a circular, convex structure after cooling down. For this void formation mechanism, continuous triangular gaps are formed along the printing direction. Furthermore, due to the addition of carbon fibre reinforcement to the PLA matrix, intra-filament voids could be formed inside the extruded composite filament. The presence short carbon fibre reinforcement can be observed under magnification, ×200. Carbon fibre reinforcements were dispersed in the PLA matrix and oriented to the printing directions, which explained the differences in stiffness of PLA-CF composites compared to PLA samples. Moreover, voids were found due to fibre pull-out during tensile testing. These results are in accordance with the findings of previous research studies [6,16,46,47,48].

#### 3.1.2. Interlaminar Shear Strength (ILSS) Performance of PLA-Based Composite Samples

**Figure 6:**
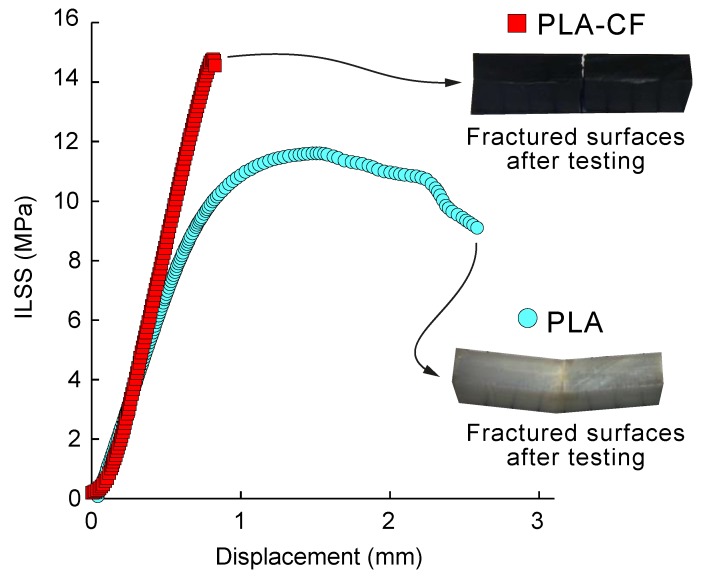
Average ILSS-displacement curves of the PLA and PLA-CF 3D printed samples. Optical micrographs showing details of the fractured surfaces after testing are included for comparative purposes.

The aim of this section was to analyse the effect of presence of carbon fibre reinforcement on the bonding performance of PLA-CF composites compared to 3D printed neat PLA samples. For this purpose, the Flat orientation was selected in this study. The best bonding performance was expected in Flat and On-edge orientations, whereas a lower interlaminar shear strength was expected in the case of the Up-right orientation. The selection of this build orientation was in accordance with previous analysis of the bonding performance in 3D printed fibre reinforced composites [6,31]. Table 5 shows average ILSS values for the neat PLA and PLA-CF composite samples, whereas Figure 6 shows a representative ILSS strength-displacement curves for the PLA and PLA composite samples.

The results showed that 3D printed PLA and PLA-CF composite samples revealed different interlaminar bonding behaviour. PLA-CF composite samples depicted the highest interlaminar shear strength and lower ductility. These results are in accordance with the behaviour reported in the flexural performance for Flat and On-edge orientations. PLA-CF specimens showed an average increase in ILSS strength of 133.30% in comparison to the PLA samples, from 11.4 MPa for PLA to 15.2 MPa for PLA-CF composites. These results were in line with previous works [6,16], and with the improved tensile and flexural behaviour exhibited by the PLA-CF composite samples and enhanced interlaminar adhesion.

### 3.2. Effect of Carbon Fibre Reinforcement and Build Orientation on the Dimensional Accuracy and Surface Texture of PLA Composite Samples

**Figure 7:**
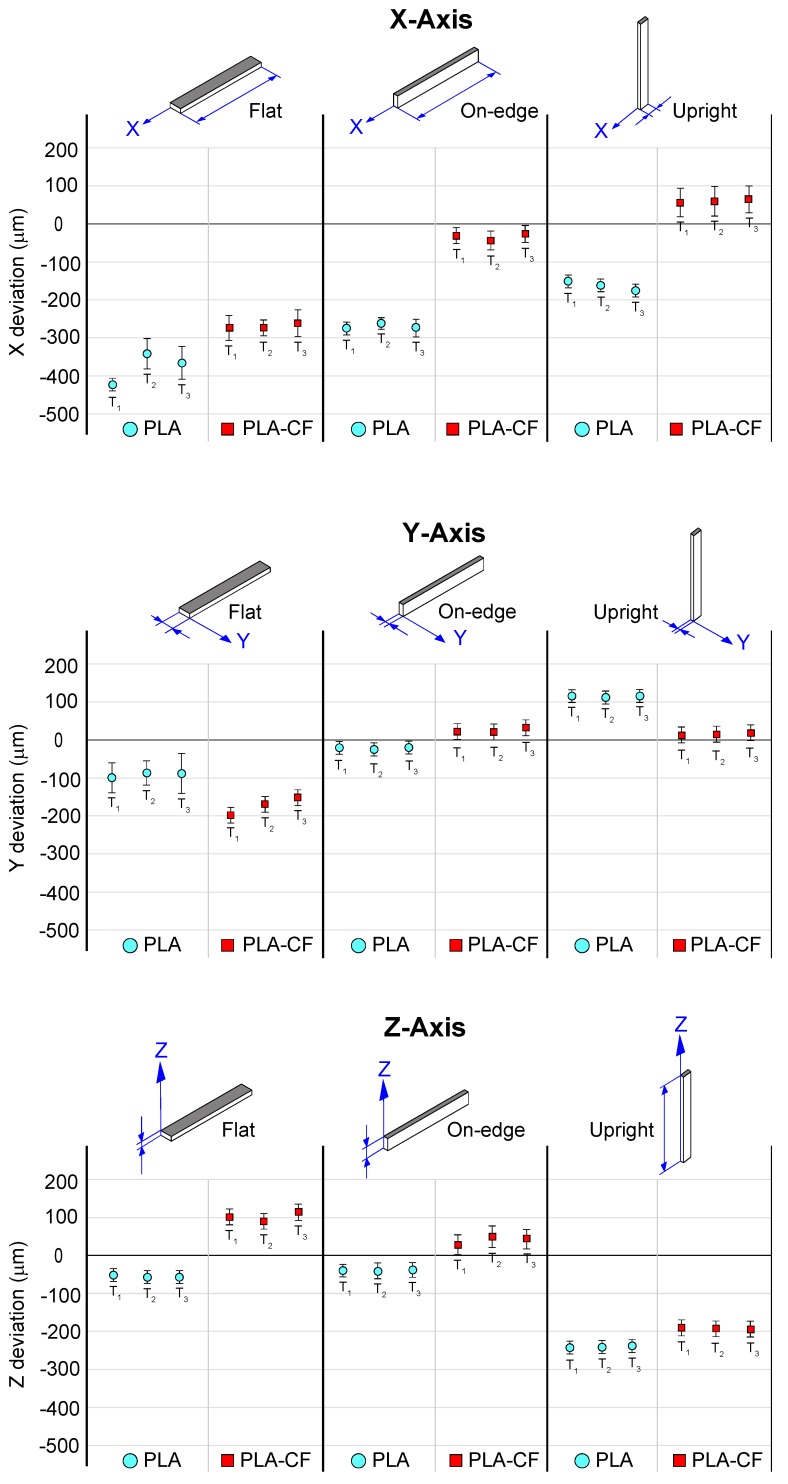
Dimensional deviation of the different PLA samples. The mean, maximum and minimum values of three samples are shown.

Table 6 and Figure 7 show the dimensional deviation (*Dx*, *Dy*, *Dz*) of PLA-base samples evaluated on the measurement axes (X, Y, Z) for each build orientation. The results of the average dimensional deviation and the standard deviation for the three samples tested (T_1_, T_2_, T_3_) of PLA and PLA-CF filaments, and the three build orientations analysed in this study are shown in Table 6. The results revealed that on the X-Y axes, the large displacement of the print head in the Flat, and On-edge orientations exhibited the greatest differences in dimensional accuracy. On the *X-*axis, Upright and On-edge orientations obtained lower dimensional deviation than the Flat orientation. In particular, Upright and On-edge PLA-CF composites showed an average deviation of 32.3 µm and 43 µm, respectively, while Flat PLA-CF composite obtained an average deviation of 266 µm. This lower dimensional deviation was expected as On-edge and Upright orientations were affected by shorter print head displacements. On the other hand, PLA samples obtained the maximum average deviation with 377 µm. On the *Y* axis, On-edge and Upright PLA-CF samples depicted lower dimensional deviation, with maximum average deviations of 26.7 µm and 20.3 µm for On-edge and Upright orientations, respectively. These results exhibited the same trend with those from X-axis. Finally, an enhancement in the dimensional accuracy of PLA samples was observed on the Z-axis, with maximum average deviations of 56 µm and 39.7 µm for Flat and On-edge orientations respectively, except in the Upright orientation owing to the higher number of accumulated layers. Moreover, the addition of carbon fibre reinforcement into the PLA matrix improved dimensional behavior in 55% PLA-CF samples, which implies that the addition of carbon fibre reinforcement, in overall terms, did not affect the dimensional accuracy, and even improved the dimensional behaviour in certain cases. In addition, the repeatability was very similar in both filament materials.

Finally, surface roughness analysis for the 3D printed PLA and PLA-CF samples was performed. The surface roughness parameter analysed in this study was the Arithmetic Mean Height (*Sa*). Moreover, the flatness deviation was obtained by the Root Mean Square Flatness Deviation (*FLTq*). Flatness and surface texture were evaluated using a sampling area of 10 × 10 mm in the X-Y plane, taking 334 profiles separated by 24 µm. The effects of the filament printing process were assessed in the Flat orientation, layer deposition in On-edge orientation and layer accumulation in the Upright orientation.

**Figure 8:**
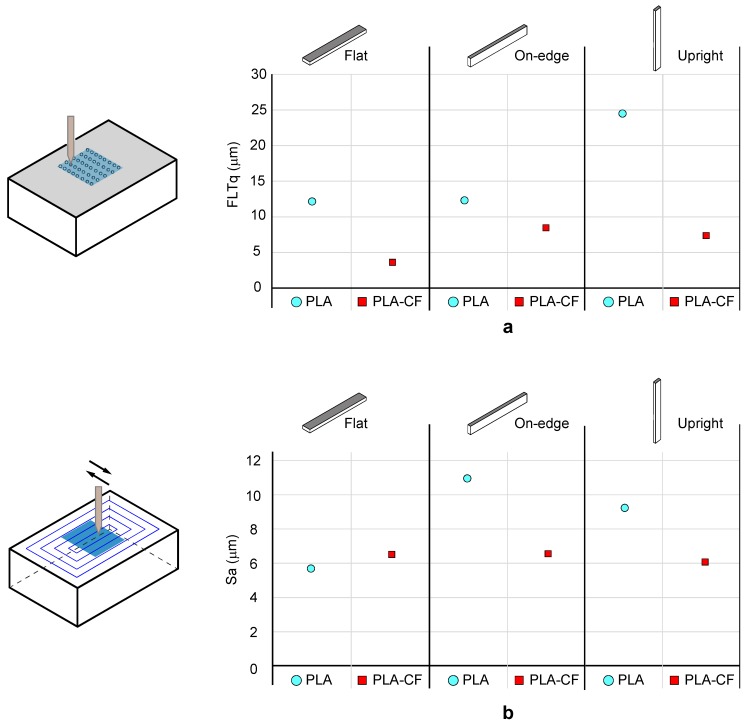
Surface quality of the different PLA samples. (**a**) Flatness deviation (*FLTq*) and (**b**) surface roughness (Sa).

In order to stablish an effective comparison in terms of surface texture, Table 7 shows the experimental data for the flatness deviation (*FLTq*), and surface roughness (*Sa*) evaluation for both the 3D PLA and PLA-CF samples. Additionally, for a better understanding, Figure 8 illustrates the graphical evolution of collected data. For the flatness deviation (Figure 8a), the addition of carbon fibres to PLA significantly improved the flatness error, in particular for the Flat and Upright orientations, where the *FLTq* parameter experimented a sharp decrease of 70%. The three build orientations exhibited good behaviour with *FLTq* values below 9 μm. As shown in Figure 8b, the addition of carbon fibres enhanced surface roughness in the On-edge and Upright orientation with a substantial reduction of 35% in the Sa parameter, whereas significant variations in the Flat position were not found. Thus, according to the results obtained, PLA-CF improved the surface roughness only on formed surfaces by accumulated layers.

## 4. Conclusions

This work analyses the effect of carbon fibre reinforcement and build orientation on the mechanical, dimensional accuracy and surface roughness performance of 3D printed PLA composite parts manufactured by fused filament fabrication technique. Two 3D printing filaments, PLA and PLA-CF reinforced with short carbon fibres, were examined. In order to determine the mechanical properties of these 3D printed samples, tensile, flexural and interlaminar shear strength tests were conducted. SEM micrographs of tensile cross-sectional tensile fractured surfaces of 3D printed PLA-CF composite samples were also evaluated.

On-edge and Flat orientations exhibited the best mechanical performance. In comparison to neat PLA, Flat PLA-CF samples depicted the greatest tensile strength and stiffness with an enhancement of 47.1% and 179.9%, respectively. Moreover, Flat PLA-CF samples showed the highest flexural strength and modulus, with an improvement of 89.75% and 230.95%, respectively, compared to the neat PLA. Furthermore, PLA-CF samples revealed the best interlaminar shear behaviour, with an average increase in ILSS strength of 133.30% compared to the neat 3D printed PLA samples. On the other hand, failure in PLA-CF composite samples occurred at lower strains than in PLA, indicating that the reinforced material was more brittle with the addition of carbon fibres.

In general, the addition of carbon fibres to the PLA matrix did not affect the dimensional accuracy of the 3D printed PLA-CF specimens. Finally, PLA-CF samples showed significant improvements in flatness error and surface texture.

In conclusion, the manufacturing of 3D printed composites with improved properties has become an interdisciplinary and cutting-edge research topic in recent years [13]. Hence, further research is needed to understand the process-property relationships and geometric characteristics of 3D printed fibre-reinforced PLA composites.

## Figures and Tables

**Table 1:** Mechanical properties of the polylactic acid (PLA)-based materials provided by the manufacturer [41,42].

Property	PLA	PLA-CF
Tensile strength (MPa)	35.6	47.9
Tensile modulus (MPa)	3420	4791
Elongation at break (%)	4.2	2.0
Flexural strength (MPa)	85.2	114
Flexural Modulus (MPa)	2378	6320
Density (g/m^3^)	1.24	1.29

**Table 2:** Ultimaker 2+ technical specifications.

Parameters	Value
Resolution	X and Y-axis =12.5 µm Z-axis = 5 µm
Temperature	Nozzle = 180–260 °C Heated bed = 20–100 °C

**Table 3:** Process variables and their levels.

Parameters	Value
Print speed (mm/s)	50
Flow rate (mm^3^/s)	4.8
Layer height (mm)	0.16
Number of top/bottom layers	5
Printing temperature (ºC)	210
Nozzle diameter (mm)	0.4
Line width (mm)	0.35
Infill pattern	Concentric
Infill density	100%

**Table 4:** Average tensile and flexural strength and stiffness for different orientations of PLA and PLA-CF samples. Standard deviation is depicted in brackets.

Orientation	Material	Tensile Results		Flexural Results	
		σ_t_ (MPa)	E_t_ (GPa)	σ_f_ (MPa)	E_f_ (GPa)
Flat	PLA	47.8 (1.1)	3.35 (0.09)	55.6 (1.5)	2.09 (0.30)
	PLA-CF	70.3 (1.3)	9.21 (0.12)	105.5 (0.4)	6.94 (0.06)
On-edge	PLA	55.7 (0.3)	3.29 (0.09)	42.5 (0.9)	1.71 (0.62)
	PLA-CF	66.1 (0.5)	8.48 (0.06)	95.7 (1.4)	6.40 (0.09)
Upright	PLA	11.5 (1.6)	3.05 (0.60)	21.3 (1.1)	1.91 (0.06)
	PLA-CF	18.2 (1.8)	3.35 (0.21)	22.0 (1.8)	2.34 (0.33)

**Table 5:** Average ILSS results of PLA and PLA-CF. Standard deviation is shown in brackets.

Interlaminar Shear Strength τILSS (MPa)	
PLA	11.4 (2.5)
PLA-CF	15.2 (1.3)

**Table 6:** Dimensional deviation $D_{\left(x,y,z\right)i}$, average ${\bar{D}}_{\left(x,y,z\right)}\;$ and standard deviation in brackets (SD) obtained for PLA and PLA-CF filaments in each build orientation and axis.

Orientation	Material	${D_{xi}}_{\;}$	${\bar{D}}_{x}(\mathrm{SD})$	$D_{yi}$	${\bar{D}}_{y}(\mathrm{SD})$	$D_{zi}$	${\bar{D}}_{z}(\mathrm{SD})$
		(μm)	(μm)	(μm)	(μm)	(μm)	(μm)
Flat	PLA	−423		−100		−52	
		−342	−377.0 (41.6)	−87	−91.7 (7.2)	−58	−56.0 (3.5)
		−366		−88		−58	
	PLA-CF	−270		−197		102	
		−270	−266.0 (6.9)	−166	−170.7 (24.3)	90	101.7 (11.5)
		−258		−149		113	
On-edge	PLA	−275		−21		−40	
		−262	−270.3 (7.2)	−25	−22.0 (2.6)	−41	−39.7 (1.5)
		−274		−20		−38	
	PLA-CF	−29		23		29	
		−42	−32.3 (8.5)	24	26.7 (5.5)	50	41.0 (10.8)
		−26		33		44	
Upright	PLA	−151		116		−242	
		−162	−163.0 (12.5)	112	115.0 (2.6)	−241	−240.3 (2.1)
		−176		117		−238	
	PLA-CF	56		15		−188	
		60	43.0 (26.1)	20	20.3 (5.5)	−190	−189.3 (1.2)
		13		26		−190	

**Table 7:** Average surface texture results as a function of build orientation of PLA and PLA-CF samples.

Orientation	Material	*FLTq*	*Sa*
		(μm)	(μm)
Flat	PLA	12.20	5.77
	PLA-CF	3.61	6.58
On-edge	PLA	12.37	10.97
	PLA-CF	8.49	6.63
Upright	PLA	24.57	9.29
	PLA-CF	7.44	6.01
